# Enhancing outcomes: neurosurgical resection in brain metastasis patients with poor Karnofsky performance score - a comprehensive survival analysis

**DOI:** 10.3389/fonc.2023.1343500

**Published:** 2024-01-10

**Authors:** Maria Goldberg, Michel G. Mondragon-Soto, Ghaith Altawalbeh, Lea Baumgart, Jens Gempt, Denise Bernhardt, Stephanie E. Combs, Bernhard Meyer, Amir Kaywan Aftahy

**Affiliations:** ^1^ Department of Neurosurgery, School of Medicine, Klinikum rechts der Isar, Technical University Munich, Munich, Germany; ^2^ Department of Neurosurgery, National Institute of Neurology and Neurosurgery, Mexico City, Mexico; ^3^ Department of Neurosurgery, University Medical Center Hamburg-Eppendorf, Hamburg, Germany; ^4^ Department of Radiation Oncology, School of Medicine, Klinikum rechts der Isar, Technical University Munich, Munich, Germany; ^5^ German Cancer Consortium (DKTK), Partner Site Munich, Munich, Germany; ^6^ Department of Radiation Sciences (DRS), Helmholtz Zentrum Munich, Institute of Innovative Radiotherapy (iRT), Munich, Germany

**Keywords:** Karnofsky performance status, neurosurgical resection, brain metastases, overall survival, systemic tumor progression

## Abstract

**Background:**

A reduced Karnofsky performance score (KPS) often leads to the discontinuation of surgical and adjuvant therapy, owing to a lack of evidence of survival and quality of life benefits. This study aimed to examine the clinical and treatment outcomes of patients with KPS < 70 after neurosurgical resection and identify prognostic factors associated with better survival.

**Methods:**

Patients with a preoperative KPS < 70 who underwent surgical resection for newly diagnosed brain metastases (BM) between 2007 and 2020 were retrospectively analyzed. The KPS, age, sex, tumor localization, cumulative tumor volume, number of lesions, extent of resection, prognostic assessment scores, adjuvant radiotherapy and systemic therapy, and presence of disease progression were analyzed. Univariate and multivariate logistic regression analyses were performed to determine the factors associated with better survival. Survival > 3 months was considered favorable and ≤ 3 months as poor.

**Results:**

A total of 140 patients were identified. Median overall survival was 5.6 months (range 0-58). There was no difference in the preoperative KPS between the groups of > 3 and ≤ 3 months (50; range, 20–60 vs. 50; range, 10–60, p = 0.077). There was a significant improvement in KPS after surgery in patients with a preoperative KPS of 20% (20 vs 40 ± 20, p = 0.048). In the other groups, no significant changes in KPS were observed. Adjuvant radiotherapy was associated with better survival (44 [84.6%] vs. 32 [36.4%]; hazard ratio [HR], 0.0363; confidence interval [CI], 0.197–0.670, p = 0.00199). Adjuvant chemotherapy and immunotherapy resulted in prolonged survival (24 [46.2%] vs. 12 [13.6%]; HR 0.474, CI 0.263–0.854, p = 0.013]. Systemic disease progression was associated with poor survival (36 [50%] vs. 71 [80.7%]; HR 5.975, CI 2.610–13.677, p < 0.001].

**Conclusion:**

Neurosurgical resection is an appropriate treatment modality for patients with low KPS. Surgery may improve functional status and facilitate further tumor-specific treatment. Combined treatment with adjuvant radiotherapy and systemic therapy was associated with improved survival in this cohort of patients. Systemic tumor progression has been identified as an independent factor for a poor prognosis. There is almost no information regarding surgical and adjuvant treatment in patients with low KPS. Our paper provides novel data on clinical outcome and survival analysis of patients with BM who underwent surgical treatment.

## 1 Introduction

The incidence of brain metastases (BM) is constantly rising, and is estimated to be approximately 100.000 new cases per year (1, 2). They are, by far, the most common brain tumors. This accelerated the development of novel systemic and local therapies (3, 4). Various treatment modalities, including surgical treatment and combined radiotherapy and chemotherapy, have significantly improved the overall survival (OS) (5–7).

Traditionally, the prognosis of patients with BM is considered extremely poor (5). Previously, many of these patients had not received curative treatment because of their reduced effectiveness and decreased survival (8–10). This has led to the development of different clinical assessment scores to identify patients who would benefit from further therapy.

Karnofsky Performance Status (KPS) (11) is one of the best-known prognostic tools for assessing patients who should undergo treatment for BM (12–14). Several prognostic models have been introduced, in which KPS is included as an independent prognostic factor for OS. Both graded prognostic assessment (GPA) and recursive partitioning analysis (RPA) have shown that KPS < 70 is associated with poor prognosis and OS < 3 months; thus, patients with KPS ≥70 would mostly be considered for further treatment (8, 15, 16).

Neurosurgical resection is a well-established treatment for BM (17, 18). The decision to perform surgical resection is based on a thorough examination of symptomatic lesions, overall KPS and patient prognosis (19). Meanwhile, the survival of patients with BM has drastically improved due to modern advances in adjuvant chemoradiotherapy and immunotherapy, changing the pattern of patient selection (20–23). In contrast, surgery can improve the survival and clinical status of patients with poor KPS, thereby facilitating further treatment (24, 25). This suggests that a low KPS does not affect the decision to perform surgery or initiate adjuvant radiotherapy or chemotherapy.

There is a big gap in the research literature regarding patients with low KPS. Most of these patients are denied the opportunity to receive the surgical treatment. Few of them receive combination treatment including neurosurgical resection (24). However, the data is missing as most of them are not being reported due to a broad consensus that surgery is not indicated in this group. Scarcity of information inhibits the development and advancement of therapy.

Therefore, it is essential to understand the clinical outcomes of patients with a low KPS (< 70) who have undergone BM surgery. Identifying the prognostic factors in this cohort of patients would help in the selection of patients who can benefit from treatment and guide the choice of therapy. This study aimed to explore the role of surgery in the improvement of clinical outcomes in patients with BM who have poor KPS and to further determine its utility in a previously overlooked cohort of patients.

## 2 Materials and methods

### 2.1 Ethical statement

This retrospective study was conducted in accordance with the ethical principles of the Declaration of Helsinki. Approval was obtained from the local ethics committee of the Technical University of Munich (no. 5626:12) and the requirement for written informed consent was waived.

### 2.2 Demographic variables

A retrospective review of institutional databases was performed and patients with KPS < 70 who underwent surgical resection for newly diagnosed BM between April 2007 and January 2020 were analyzed. Exclusion criteria were as follows: previous treatment for BM, biopsy, or missing data regarding postoperative treatment. All our patients had precocious and synchronous BM, so the surgery was prioritized before the beginning of adjuvant treatment in all patients independent of neurological symptoms. Metachronous BM were not included in further analysis. The KPS, on a scale of 0 to 100, with 100 representing the best status, was used to evaluate the quality of life and patients’ physical condition before and shortly after surgery during the same hospital stay. Patients were then grouped based on their KPS value. The groups were compared separably.

The following information was extracted from the electronic medical records: age at diagnosis, sex, tumor localization, preoperative tumor volume, number of BM, complete tumor resection, adjuvant therapy, systemic progression on therapy, and date of death or loss to follow-up. The decision again the adjuvant therapy was based on low functional status and family’s decision made toward palliative treatment. OS was also analyzed. Based on clinical characteristics, the pre- and postoperative RPA and Eastern Cooperative Oncology Group (ECOG) performance status scores were calculated.

### 2.3 Treatment

Recommendations for surgical treatment were made by an interdisciplinary oncological board based on the presence of a new neurological deficit, tumor mass effect, intratumoral hemorrhage, large lesions in the posterior fossa, and an unknown histology. Epileptic seizures and motor deficits associated with brain edema, even in patients with extremely small lesions, were an indication for surgical resection. None of the patients received biopsy. Neurosurgical resection was performed using various intraoperative neuromonitoring techniques to achieve maximal tumor removal. Postoperative MRI was performed within 72 h of the surgical procedure. The T1-weighted images were analyzed using Origin^®^ Software (Brainlab, Ver 3.1, Brainlab AG, Munich, Germany), where any contrast-enhancing lesions were determined as tumor rests. The decision for subsequent systemic therapy and radiotherapy was made by the interdisciplinary oncological board based on the clinical status, histopathological results, extent of resection, and the patient’s choice. All patients included in our analysis were initially treatment naïve.

### 2.4 Statistical analysis

Statistical analyses were performed using SPSS (version 29.0.1.0; IBM, Chicago, IL, USA) and GraphPad Prism (version 8.3.1; La Jolla, CA, USA). In terms of prognosis, OS > 3 months after surgery was considered favorable, whereas OS ≤ 3 months was considered poor. According to the literature patients with extremely low clinical status show survival < 3 months. So, the cutoff 3 months was chosen to define individuals who lived longer than expected based on the current data (8, 15, 16). Recently, Park et el (26). suggested a model for survival of more and less than 3 months in order to identify the patients with low KPS who show better survival and who need further treatment. We found this grouping appropriate in this cohort of patients and tried to identify differences within these groups. The KPS were compared using the Wilcoxon test. Univariate analysis was performed to identify the risk factors that could influence survival after neurosurgical resection. Multivariate Cox regression analysis was used for previously reported significant variables. The Kaplan–Meier method was used to perform survival analysis. Statistical tests were two-sided, and p-values < 0.05 were considered significant.

## 3 Results

### 3.1 Demographic data

A total of 140 patients with a KPS of < 70 who underwent surgical resection of BM between 2007 and 2020 met the inclusion criteria, death of 102 patients was documented. The demographic characteristics are summarized in **Table 1**. The median age at the time of the first BM diagnosis was 66.1 years (range, 33–93 years). Sex distribution was equal, with 73 (52%) male and 67 (48%) female patients. Primary histology comprised non-small cell lung cancer (n = 66, 47.1%), breast cancer (n = 21, 15%), gastrointestinal tumors (n = 13, 9.3%), renal cell carcinoma (n = 9, 6.4%), prostatic adenocarcinoma (n = 7, 5%), cancer of unknown primary (n = 6, 4.4%), small cell lung cancer (n = 5, 3.6%), ovarian cancer (n = 4, 2.9%), thyroid cancer (n =3, 2.1%), endometrial cancer (n = 2, 1.4%), squamous cell carcinoma (n = 2, 1.4%), chondrosarcoma (n = 1, 0.7%), and sarcoma (n = 1, 0.7%). Regarding the number of ontracranial lesions, 73 (52.1%), 20 (14.3%), 30 (21.5%), and 17 (12.1%) patients had 1, 2, 3, and >3 intracranial lesions, respectively. A total of 111 (79.3%) lesions were supratentorial and 29 (20.7%) were infratentorial. Median preoperative tumor volume was 26.06 cm^3^ (range, 0.29–94.3). Complete resection was achieved in 83 (59.4%) patients; 77 (55%) underwent postoperative radiotherapy, 34 (24.3%) received adjuvant systemic chemotherapy or immunotherapy, and 32 (22.8%) showed systemic disease progression.

**Table 1:** Demographic and baseline characteristics of the patients.

Parameter	Value
**Age (median, range)**	66.1 (33–93)
**Sex (n, %)**	F 67 (48%) M 73 (52%)
**KPS preoperative (%, range)** **KPS postoperative (%, range)**	50 (10-60) 50 (10–100)
**ECOG preop (score unit, range)** **ECOG postop (score unit, range)**	3 (2–4) 3 (0–4)
Histology (n, %)	
**NSCLC** **Breast cancer** **GI tumor** **RCC** **Prostatic cancer** **CUP** **Other**	66 (47.1%) 21 (15%) 13 (9.3%) 9 (6.4%) 7 (5%) 6 (4.4%) 18 (12.8%)
Tumor localization (n, %)	
**Supratentorial** **Infratentorial**	111 (79.3%) 29 (20.7%)
Number or BM (n, %)	
**1** **2** **3** **>3**	73 (52.1%) 20 (14.3%) 30 (21.5%) 17 (12.1%)
**Preoperative tumor volume (median, range)**	26.06 cm^3^ (0.29–94.3)
**Complete resection (n, %)**	83 (59.4%)
Postoperative radiotherapy (n, %)	
**WBRT** **SRS** **HSRS** **None**	37 (26.4%) 3 (2.2%) 37 (26.4%) 63 (45%)
Postoperative systemic therapy	
**Chemo- Immunotherapy** **None**	34 (24.3%) 106 (75.7)
**Systemic progression (n, %)**	32 (22.8%)

KPS, Karnofsky performance scale; ECOG, Eastern Cooperative Oncology Group status; NSCLC, Non-small cell lung cancer; GI, gastrointestinal cancer; RCC, renal cell carcinoma; CUP, Cancer of Unknown Primary; BM, brain metastases; WBRT, whole brain radiation therapy; SRS, stereotactic radiosurgery, (HSRS) hypofractionated stereotactic radiosurgery.

### 3.2 Clinical outcomes

**Figure 1:**
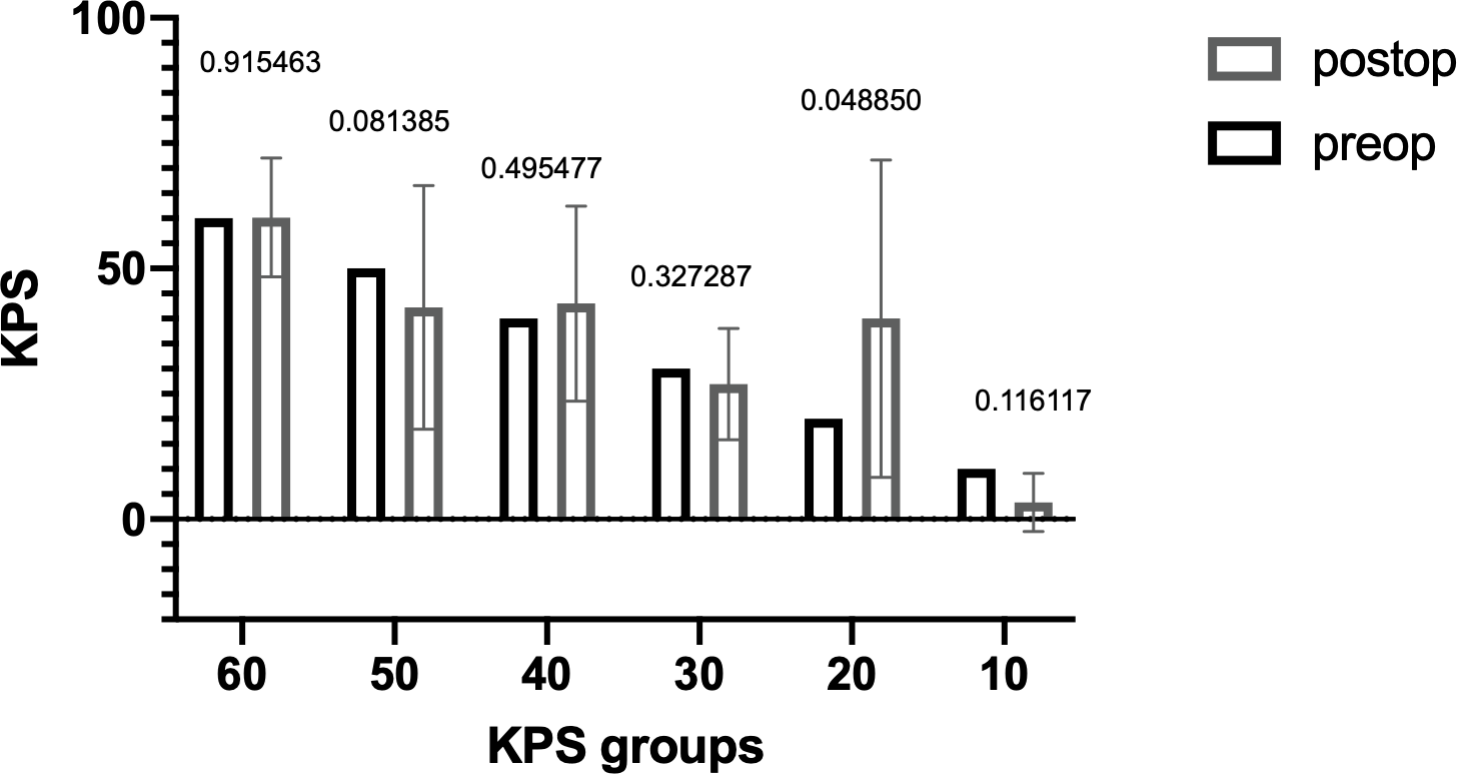
Median overall survival of all patients with brain metastasis and Karnofsky Performance Score < 70 who underwent surgery.

**Figure 2:**
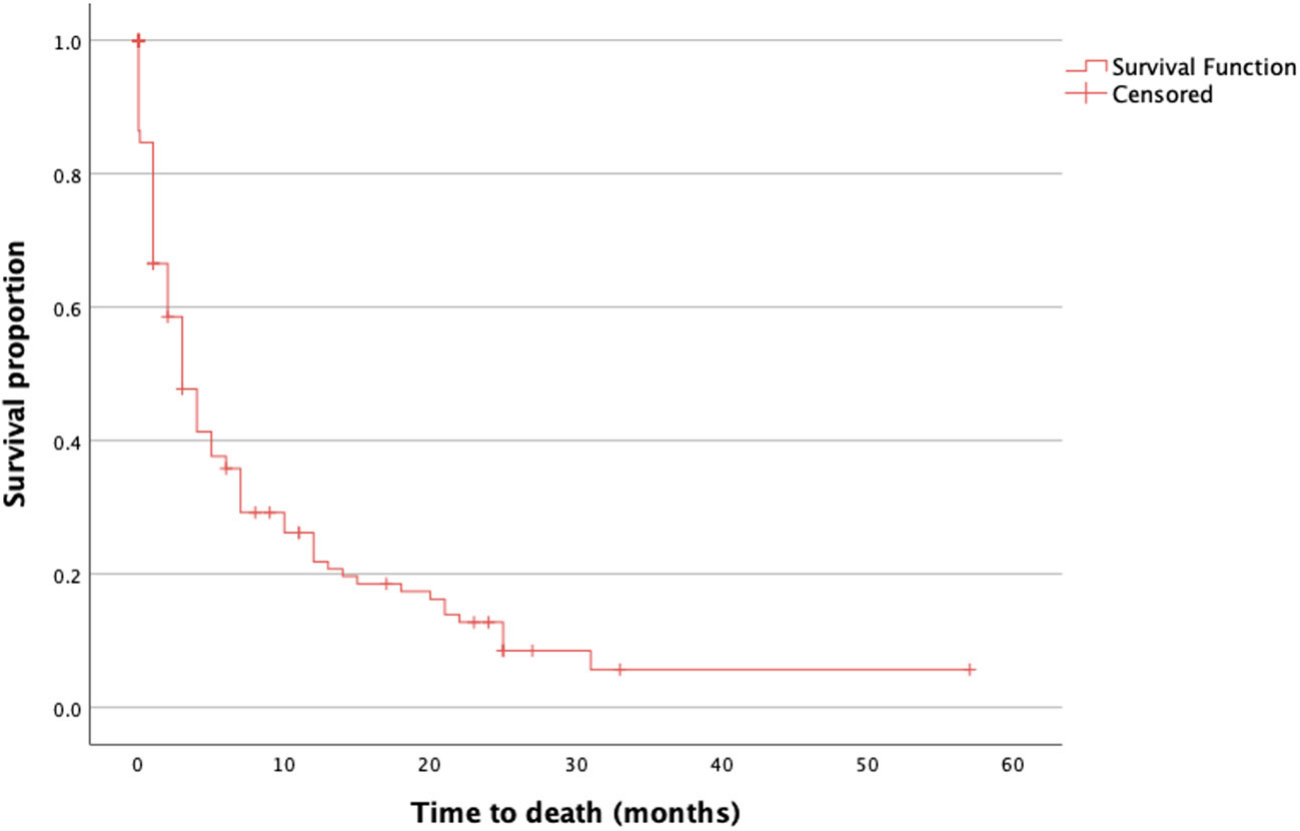
Distribution of KPS before and after surgery. The difference in KPS before (black) and after (gray) surgery is shown as the mean with SD. The Wilcoxon signed-rank test was also performed. P-values are shown for each group. There was a significant difference in the postoperative KPS in the group with an initial KPS of 20% (p = 0.048). KPS, Karnofsky Performance Score.

The median OS was 5.6 months (range, 0–58 months) with 52 (37.1%) patients having an OS > 3 months (**Figure 1**). The median preoperative KPS was 50% (range, 10–60). The median postoperative KPS was 50% (range, 10–100). There was a significant improvement in the clinical status in the group of patients with preoperative KPS of 20% (**Figure 2**, preoperative, 20 vs. mean postoperative, 40 ± 20, p = 0.048). There was no significant difference in the mean ECOG performance status scores (3, range 2–4 vs. 3; range, 0–5) scores after surgical treatment. There was no difference in the preoperative KPS between the two groups, >3 and ≤ 3 months respectively (50; range, 20–60 vs. 50; range, 10–60, p = 0.077).

Several patients showed deterioration of KPS, so we further analyzed surgical complication in the whole cohort. Among 22 patients, who’s KPS reduced after surgery 11 (50%) developed intraoperative or postoperative complications (4 (18.2%) patients underwent a second surgery due to postoperative intracranial hemorrhage or experienced extreme intraoperative blood lost, 3 (13.6%) patients developed hydrocephalus, 2 (9.1%) meningitis, 2 (9.1%) uncontrolled intracranial pressure increase due to cerebral edema). 1 (4.5%) patient developed acute kidney injury shortly after surgery and 1 (4.5%) patient showed pulmonary embolism. Reduction in other 9 (41%) patients was associated with chronic heart or kidney disease or tumor systemic progression. Among 118 patients who showed stable or improved KPS postoperatively 16 (13.6%) experienced postoperative complications (3 (2.5%) meningitis, 2 (1.7%) CSF Leak, 2 (1.7%) hemorrhage, 2 (1.7%) stroke, 7 (6%) wound infection).

Postoperative radiotherapy (hazard ratio [HR], 0.255; confidence interval [CI], 0.164–0.396, p < 0.001) and systemic therapy (HR 0.404; CI 0.235–0.695, p = 0.001) were the prognostic factors associated with favorable survival. In the better survival and poorer prognosis groups, 44 (84.6%) and 32 (36.4%) patients received adjuvant radiation, respectively. Systemic disease progression was another prognostic factor for poor survival (HR 3.638; CI 1.979–6.687, p < 0.001) (**Table 2**). Seventy-one (80.7%) patients in the group with an OS ≤ 3 months showed disease progression, whereas only 36 (50%) patients in the group with an OS > 3 months presented with systemic progression. Preoperative tumor volume, anatomical localization, number of lesions, and the extent of resection did not affect survival.

**Table 2:** Univariate logistic regression of demographic factors associated with favorable and poor prognosis.

Variable	> 3 months (n = 52)	≤ 3 months (n = 88)	HR	95% CI lower	95% CI upper	P value
**Age (range)**	64+-11.67 (37–83)	67 +-11.32 (33–93)	1.01	.975	1.025	.980
**Female (%)**	28 (53.8%)	40 (45.5%)	1.119	.640	1.955	.693
**KPS preoperative**	53 +-11.47 (20–60)	45.45 +- 14.11 (10–60)	1.002	.978	.1.026	.891
**ECOG preoperative**	2.52 +- 0.66 (2–4)	2.92 +- 0.81 (2–4)	.952	.894	1.014	.126
**RPA preop**	3 (3–3)	3 (3–3)	.957	.901	1.016	.147
**GPA preop**	1.12 +- 0.7 (0–3)	0.98 +-0.59 (0–3)	.838	.617	1.137	.257
**Tumor volume preoperative cm^3^**	23.05 +-18.55 (0.1–67.99)	22.37 +- 20.43 (0.41–94.31)	.996	.987	1.005	.415
**Postoperative radiotherapy**	**44 (84.6%)**	**32 (36.4%)**	**.255**	**.164**	**.396**	**<.001**
**Postoperative chemo-immunotherapy**	**24 (46.2%)**	**12 (13.6%)**	**.404**	**.235**	**.695**	**.001**
**Number of BM**	2.4 +-2.2 (1–10)	2.1 +-2.03 (1–15)	1.034	.980	1.091	.226
**Localization supra-infratentorial**	44 (84.6%)	66 (75.0%)	.493	.515	1.376	.493
**Complete resection**	33 (63.5%)	50 (56.8%)	.919	.620	1.364	.676
**Systemic progression**	**36 (50%)**	**71 (80.7%)**	**3.638**	**1.979**	**6.687**	**<.001**

KPS, Karnofsky Performance Score; ECOG, Eastern Cooperative Oncology Group status; RPA, recursive partitioning analysis; GPA, graded prognostic assessment; BM, brain metastasis; HR, hazard ratio; CI, confidence interval.

Postoperative radiotherapy, and Postoperative chemo-immunotherapy were associated to an Overall Survival >3 months in a statistically significant fashion.

Systemic progession was significantly associated with an. overall survival below 3 months.

In the multivariate Cox hazard regression test, adjuvant radiotherapy (HR 0.0363; CI 0.197–0.670, p = 0.001), adjuvant chemotherapy and immunotherapy (HR 0.474; CI 0.263–0.854, p = 0.013), and absence of systemic disease progression (HR 5.975; CI 2.610–13.677, p < 0.001) were independent factors associated with better survival (**Table 3**).

**Table 3:** Multivariate Cox regression analysis including previously identified prognostic factors.

Variable	HR	95% CI lower	95% CI upper	P value
**Adjuvant radiotherapy**	.363	.197	.670	.001
**Adjuvant chemo- immunotherapy**	.474	.263	.854	.013
**Systemic progression**	5.975	2.610	13.677	<.001

HR, hazard ratio; CI, confidence interval.

**Figure 3:**
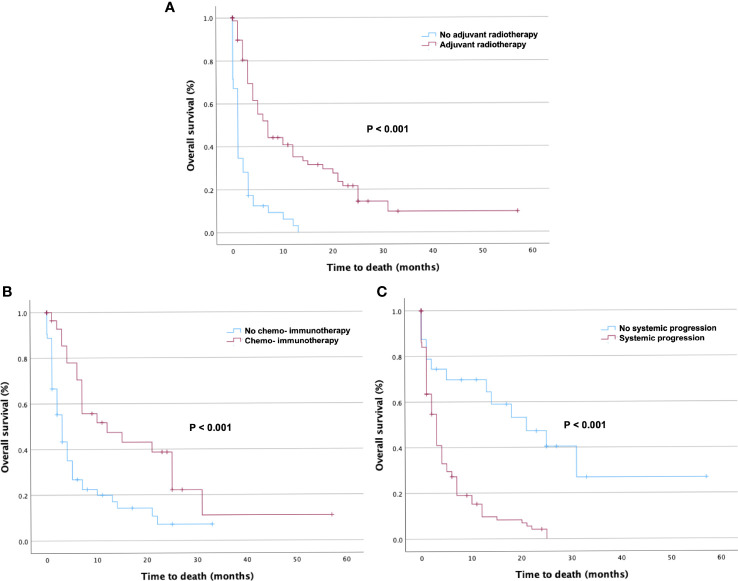
Kaplan–Meier curves of overall survival (OS) in patients stratified by prognostic factors. Kaplan–Meier curves of OS in patients with Karnofsky Performance Score < 70 who underwent surgical resection stratified by **(A)** adjuvant radiotherapy, **(B)** adjuvant chemotherapy and immunotherapy, and **(C)** presence of systemic disease progression. The p-values of the log-rank tests are shown.

Kaplan–Meier curves were used to present the distribution of OS for adjuvant radiotherapy (**Figure 3A**), adjuvant chemotherapy and immunotherapy (**Figure 3B**), and systemic disease progression (**Figure 3C**).

## 4 Discussion

This single-center retrospective study sought to investigate the potential role of surgical treatment in a cohort of patients with a low KPS. Neurological deterioration, which often accompanies intracranial metastases, rapidly leads to altered mental status and reduced clinical performance, worsening the KPS (27). A low KPS predicts poor prognosis and worse treatment tolerance, preventing recruitment and leading to the exclusion of these patients from prospective clinical trials (28, 29). Moreover, treatment options for these patients are commonly restricted to palliative and supportive care (30). Historically, poor KPS has been a key factor in clinical decision-making, hindering further tumor-specific treatment (31). Therefore, this cohort of patients remains understudied and information on management recommendations is limited. Advances in local and systemic treatment options may facilitate therapy for patients with KPS < 70.

The OS of patients with BM and a low KPS after treatment initiation varies in the literature; it is approximated to range from less than 4 months (16, 32–34) to 10 months. In a recent report, an even higher OS rate was recorded (20, 24).

Surgical treatment can rapidly improve focal neurological symptoms and the overall status by relieving the symptoms of intracranial hypertension (35). Moreover, changes in the molecular profile of the primary tumor established another important role of surgical resection in obtaining histopathological samples for further analysis (36). Maximal cytoreduction is an independent prognostic factor in the treatment of BM (37–39).

An OS of 5.6 months after surgery was reported, with high variability (range, 0–58 months) within the group. This highlights the importance of identifying the factors associated with prolonged survival and selecting appropriate candidates for maximal treatment. A cutoff OS of > 3 months was defined as favorable, as described previously (40). Factors associated with the 3-months survival after surgical resection were analyzed.

Some factors associated with a favorable OS have been shown to be significant. For instance, surgical resection has been shown to improve the KPS, which, in turn, increases the chances of receiving effective adjuvant therapy (20). However, this is only applicable to patients with higher KPS. Moreover, surgery showed survival benefits for patients with a KPS < 70 compared with patients who did not receive surgical treatment (24). In this study, a significant improvement was observed in the group of patients with a preoperative KPS of 20, which supports the importance of surgery in combination therapy for BM. No difference in postoperative KPS was observed between the other groups, indicating that surgery is an appropriate option despite the high rate of perioperative morbidity (26). No factors that would make surgery beneficial in different KPS groups were identified. Our study group believes that, in general, the association of KPS deterioration with intracranial lesions and rapid worsening of neurological symptoms prior to surgery could explain fast improvement afterwards. This could be secondary to several reasons: the presence of intracranial hypertension due to the mass effect arising from both edema and the metastatic lesion, changes in the cerebral microenvironment aroused by the presence of a different histology, possible changes in the brain hemodynamics, subjacent epileptogenic activity, as well as the interactions between medication (antiepileptic drugs, steroids, chemotherapy, etc.) (20, 25).

Various clinical and treatment-related variables are associated with improved survival rates. Cumulative tumor volume is reportedly associated with survival benefits (1, 41). These findings were confirmed in a study by Park et al. (40); however, their analysis focused on a single histological entity. In the current study, no association was observed with preoperative tumor volume. Disease progression is a well-known prognostic factor that is associated with poor survival (42, 43). Systemic progression during ongoing therapy was confirmed as an independent prognostic marker of shorter OS in the present study.

Compared with radiation alone, combined neurosurgical resection and radiotherapy were not found to be beneficial when the patients were not selected based on their clinical status and systemic progression (44). Furthermore, adjuvant radiotherapy is an effective method for local tumor control (45). In our cohort, adjuvant radiotherapy was associated with improved OS. Systemic adjuvant treatment with chemotherapeutic drugs and immune system modulators is believed to be beneficial for the treatment of BM (46–49). Analysis of the data demonstrated that adjuvant systemic therapy was associated with survival benefits compared to patients who did not undergo this treatment. Although, importance of radiotherapy and systemic treatment has been shown before, the current study highlights the necessity of these treatments in patients with poor functional status in addition to offering surgical treatment as an option for alleviating neurological symptoms.

A low KPS is associated with poor survival and clinical outcomes in patients with BM. However, this should not hinder the choice of palliative or tumor-specific treatment. Current data provides a valuable information for the patients and their families regarding prognosis and risks after surgical treatment. The risks are high, but the statement that the risk of clinical deterioration postoperatively and subsequent failure of adjuvant treatment is higher in this cohort is wrong. Surgery gives an opportunity to improve neurological symptoms and KPS which may increase the chances of good treatment response. Neurosurgical resection does not deteriorate functional status and, in combination with adjuvant radiotherapy and systemic treatment, improves overall survival. Identification of new prognostic markers is essential for appropriate patient selection and prognostic evaluation.

### 4.1 Limitations

The limitations of our study include its retrospective design, the moderate size of the cohort, and the various histopathological profiles of the tumors treated at our center. The stratification of patients into different pathologies and treatment regimens for each one is limited due to the small number of patients with brain metastases and low KPS. It is difficult to make a single statement regarding the treatment regimens for all the spectrum of pathologies that comprise the oncologic diseases with brain metastases. Nowadays, driver mutations and immune checkpoint expression are extremely relevant for interpretation of treatment-related outcomes. Molecular analysis was not part of routine diagnostics in the early years. We, unfortunately, cannot provide the mutation and expression status for all our patients. Not only the heterogeneity in tumor histology and treatment modalities, but also anatomical localization could further complicate data interpretation. Our study focused exclusively on surgically treated patients with initially rapid KPS deterioration preoperatively associated with BM. Comparison with relapsed patients would strengthen the study.

However, the lack of information about the role of surgery in patients with BM and low KPS makes the data remarkably relevant for decision-making in patients with advanced stages of oncological disease.

### 4.2 Conclusion

Surgical resection of singular or multiple BM can be considered as an efficient treatment modality in patients with low KPS. Surgery may improve functional status and facilitate further tumor-specific treatment even despite the possible surgical complications. Combined treatment with adjuvant radiotherapy and systemic therapy was associated with improved survival in this cohort of patients. Systemic tumor progression has been identified as an independent factor of poor prognosis.

The current study suggests that the benefit of initial surgical resection on the clinical outcome and OS in patients with BM is significant. Our findings challenge the current paradigm of BM management, raising the opportunity to perform further randomized clinical trials to investigate the role of initial systematic surgical resection of BM in patients with low KPS.

## Data availability statement

The raw data supporting the conclusions of this article will be made available by the authors, without undue reservation.

## Ethics statement

The studies involving humans were approved by ethics committee of the Technical University of Munich (no. 5626:12). The studies were conducted in accordance with the local legislation and institutional requirements. Written informed consent for participation was not required from the participants or the participants’ legal guardians/next of kin in accordance with the national legislation and institutional requirements.

## Author contributions

MG: Formal analysis, Investigation, Methodology, Supervision, Writing – original draft, Writing – review & editing. MM: Writing – original draft, Writing – review & editing. GA: Writing – original draft, Writing – review & editing. LB: Writing – original draft, Writing – review & editing. JG: Writing – original draft, Writing – review & editing. DB: Conceptualization, Investigation, Writing – original draft, Writing – review & editing. SC: Writing – original draft, Writing – review & editing. BM: Supervision, Writing – original draft, Writing – review & editing. AA: Data curation, Project administration, Supervision, Writing – original draft, Writing – review & editing.
